# Medical Missionaries

**Published:** 1888-05

**Authors:** 


### SELECTIONS.

### Medical Missionaries.

In very many of the missions for the spread of evangelical Christianity among heathen nations, the physician is regarded as almost as essential as the clergyman, and it is through the good results of the former's ministrations that the latter is enabled to gain a hearing. This is an eminently practical way of reaching the heathen, and to it is doubtless due the greater part of whatever success these missions have had. There ought to be little difficulty in securing medical missionaries enough to supply the demand. There is little attraction, for a young graduate without means or unusual abilities, in the prospect of a long struggle after a practice sufficient to support him in even comparative comfort in civilized countries. But the thought of a journey to distant lands, plenty of congenial work to do, and a support from the start, is pleasing to many even who may be but little influenced by the religious aspect of their calling. And when to this is added the powerful attraction of religious work to a truly pious mind, the impulse to offer one's self for the mission is well nigh irresistible. But it is well to look at the dark side of such a life before entering upon it, for after the work is once begun it can not be laid down lightly and without good cause. A medical missionary should, above all, be a thoroughly educated physician, not merely a graduate who has passed a creditable examination and has attended a few clinics. He should be one who has studied disease practically as a hospital interne, and who is competent to treat any affection of any organ—the eye, the throat, and the ear, as well as the heart, the lungs, or the stomach. He should, of course, be thoroughly in sympathy with the religious work of the mission, for he is to be a missionary as well as a physician; indeed the primary reason of his going is to pave the way for the conversion of the people among whom he is to live. Finally, he should not only be healthy, but of robust physique and iron constitution. He is going to a new climate, and one that is often most trying, if not deadly, to people from temperate regions, and he will be, by reason of his work, more exposed than any others of his companions, to the dangers of this climate. Then, again, the medical missionary is sometimes exposed to considerable personal danger at the hands of ungrateful patients, or of the ungrateful friends of patients. The heathen are no better than other people. When a case goes wrong they abuse the physician just as roundly as good christians do, and their abuse is apt to be even more active and to express itself by the attempt to inflict bodily injury rather than damage to reputation and good name. Medical missionaries in China and elsewhere have been forced to flee for their lives to escape the fury of an angry mob of the friends of some patient who had unfortunately died after an operation. We have no desire to discourage young men or women from embracing the calling of a medical missionary, but it is better to put the facts plainly before them while they are still here, than it is for them to learn them after they have journeyed thousands of miles and have found themselves in a position from which they can not honorably retire. The medical missionary societies do all they can to put the matter in a proper light before their candidates, but it is hard to restrain enthusiasts who believe that they have a vocation, from embracing it, in despite of any and all obstacles which may be thrown in their way.

